# Play the plug: How bacteria modify recognition by host receptors?

**DOI:** 10.3389/fmicb.2022.960326

**Published:** 2022-10-14

**Authors:** Suma Tiruvayipati, Dharjath S. Hameed, Niyaz Ahmed

**Affiliations:** ^1^Infectious Diseases Programme, Department of Medicine, Yong Loo Lin School of Medicine, National University of Singapore, Singapore; ^2^Department of Chemical Immunology, Leiden University Medical Center, Leiden, Netherlands; ^3^Pathogen Biology Laboratory, Department of Biotechnology and Bioinformatics, University of Hyderabad, Hyderabad, India

**Keywords:** bacteria host interaction, receptor modification, microbiota, antimicrobial resistance (AMR), infection and immunity

## Abstract

The diverse microbial community that colonizes the gastrointestinal tract has remarkable effects on the host immune system and physiology resulting in homeostasis or disease. In both scenarios, the gut microbiota interacts with their host through ligand-receptor binding whereby the downstream signaling processes determine the outcome of the interaction as disease or the counteractive immune responses of the host. Despite several studies on microbe-host interactions and the mechanisms by which this intricate process happens, a comprehensive and updated inventory of known ligand-receptor interactions and their roles in disease is paramount. The ligands which originate as a result of microbial responses to the host environment contribute to either symbiotic or parasitic relationships. On the other hand, the host receptors counteract the ligand actions by mounting a neutral or an innate response. The varying degrees of polymorphic changes in the host receptors contribute to specificity of interaction with the microbial ligands. Additionally, pathogenic microbes manipulate host receptors with endogenous enzymes belonging to the effector protein family. This review focuses on the diversity and similarity in the gut microbiome-host interactions both in health and disease conditions. It thus establishes an overview that can help identify potential therapeutic targets in response to critically soaring antimicrobial resistance as juxtaposed to tardy antibiotic development research.

## Introduction

Facultative anaerobic enteric bacteria form a part of the normal intestinal microbiota of humans and animals (Morales-López et al., 2019; Dougnon et al., 2020; Janda and Abbott, 2021). These bacteria which mostly belong to the Enterobacteriaceae family are responsible for shaping both the metabolic and physiological processes in addition to the development of the immune system in the host body (Martin et al., 2019; Bajinka et al., 2020). A majority of gastrointestinal (GI) microbes form a complex bacterial community and maintain a commensal relationship with the host. Some bacterial species of the genera *Escherichia*, *Shigella*, *Salmonella*, *Proteus* and *Yersinia* constantly invade the host GI tract, the blood and vascular system and the genitourinary tract thereby causing and spreading infection (Dekker and Frank, 2015; Hamilton et al., 2018; Gu et al., 2019; Aljahdali et al., 2020; Ramos et al., 2020; Yang et al., 2020). Among various defense mechanisms, the formation of a mucus layer by the host cells in the gut acts as a natural barrier blocking foreign pathogens from invading the intestinal epithelial lining. This mucus membrane acts as a major component of the innate immunity (Okumura and Takeda, 2017; Sicard et al., 2017). A large number of intestinal microbes inhabit the outer mucus layer. The constant degradation and replenishment of the mucin layer helps resist colonization and promote commensalism with the gut microbiota (Josenhans et al., 2020; Fang et al., 2021). Nevertheless, invasive bacterial strains residing in the intestine have evolved strategies to adhere to or swim out of the mucus barrier by the production of adhesins, flagella, and fimbriae, leading to a chronic and sometimes symptomatic colonization in the gut. A compromised mucus barrier can lead to various pathological conditions (Cornick et al., 2015; Paone and Cani, 2020). In response to mucin degradation, the host responds with a myriad of defensive measures against the microbial attack. Among them is the escalation of the host immune response through recognition of pathogen- or damage-associated molecular patterns (PAMPs or DAMPs) by the pattern recognition receptors (PRRs). The PAMPs can be polymers of carbohydrates, proteins or even nucleic acids that are essential for microbial survival and hence their modifications could be detrimental (Patten and Collett, 2013). The PRRs are specialized immune receptors on the host epithelial cells which act upon binding with microbial PAMPs. The most abundant PRRs are the toll-like receptors (TLRs) that are germ line-encoded in the immune system (Akira et al., 2006; Nie et al., 2018). Downstream to PAMP–PRR interaction both on the cell surface or in the intracellular environment, several proinflammatory and anti-microbial responses are triggered by the activation of a wide range of intracellular signaling pathways (Akira et al., 2006). Phagocytic and antigen-presenting cells (APCs) then mediate the innate immune responses. Furthermore, the PRR-induced signaling pathways result in the synthesis of molecules such as chemokines, cytokines, cell adhesion molecules, and immunoreceptors underpinning an early host response to infection (Iwasaki and Medzhitov, 2004; Kogut et al., 2020). To counteract reactions elicited by these receptors, microbes express effector proteins which have the ability to perturb host defense mechanisms. These effector proteins facilitate microbial infection whilst deriving nutrients from the host (Santos, 2015; Popa et al., 2016). In turn, the immune system monitoring the microbial communities acts by inducing inflammation against potential pathogens while retaining tolerance towards commensals in the gut (Tel et al., 2016; Zheng et al., 2020). To this end, many studies have reported on how different microbial ligands modulate microbial sensing of the host. While studies have focused rather exclusively on a few host receptors, a summarized information on the diversity of the host receptors and microbial ligands is limited. This review focuses on consolidating the diversity of enterobacterial interactions with microbial sensors to gain an understanding on how bacteria could possibly modify recognition by host receptors. Furthermore, a brief outlook into the potential therapeutic values of the host-microbiome interactions and the players involved in this process are also addressed.

### Human host receptors

The gut mucosa has the ability to readily identify and neutralize microbial infections in addition to distinguishing a microbial attack by pathogens from symbiotic relationship of non-pathogens. Human host receptors of the innate immune system, namely the toll-like receptors (TLRs), nucleotide-binding and oligomerization domain (NOD)-like receptors (NLRs), C-type lectin receptors (CTLRs) and the retinoic acid-inducible gene I (RIG-I)-like receptors (RLRs) play a crucial role in host-microbiota communication (Delbridge and O’Riordan, 2007; Kawai and Akira, 2011; Dierking and Pita, 2020). These receptors differ from two other PRRs, i.e., G-protein coupled receptors (GPCRs) and peptidoglycan (PG) recognition proteins (PGRPs) by their specificity to recognize distinct microbial molecules (Dierking and Pita, 2020). The specificity with which PRRs bind with the ligands from pathogenic microbes (PAMPs) are based on different molecular structures and composition of these ligands. Furthermore, different structures of these PAMPs are essential for microbial viability and are specific to microorganisms. Thus, the host PRRs have evolved to distinctly communicate with commensal microbes and pathogens (Chu and Mazmanian, 2013; Li and Wu, 2021).

#### Toll-like receptors and NOD-like receptors

Among the aforementioned receptors, the TLRs and NLRs distinctly recognize microbe-associated molecular patterns (MAMPs) which are generally expressed by the resident microbes, and the PAMPs usually expressed by the invasive microbes (Gao et al., 2017; Negi et al., 2019). Upon recognition of these molecular patterns, the TLRs trigger signaling from the surface of the cell or endosomes, whereas the NLRs are activated in the cytosol by molecules derived from bacteria, such as flagellins, toxins, RNA and PG (Delbridge and O’Riordan, 2007).

Functionally, the TLRs are categorized into cell membrane TLRs and intracellular TLRs (nucleic acid sensors). For example, the heterodimers of TLR2 in conjunction with TLR1 or TLR6 besides TLR4, TLR5, and TLR10, express on the cell surface and belong to the cell membrane TLRs category (Gay et al., 2014). Whereas TLR3, TLR7, TLR8, and TLR9 which are localized to the intracellular compartments of the cell such as endosomes and lysosomes are the intracellular TLRs (Kawasaki and Kawai, 2014; Sellge and Kufer, 2015). TLRs produce pro-inflammatory cytokines upon binding with PAMPs (Vidya et al., 2018). The TLR is activated by PAMPs, leading to nuclear translocation of a pro-inflammatory transcription factor called activator protein 1 (AP-1), interferon regulatory factor (IRF) 3, and the nuclear factor kappa-B (NF-kB) by the recruitment of adapter proteins. These adapter proteins subsequently recruit interleukin-1R (IL-1R)-associated protein kinases (IRAK) 1, 2, 4, M, transforming growth factor-β-activated kinase (TAK) 1-binding proteins (TAB) 2 and tumor necrosis factor receptor-associated factor 6 (TRAF6; El-Zayat et al., 2019). Specific genes are then transcribed by these transcription factors encoding a different set of proteins such as chemokines [C-X-C motif chemokine ligand (CXCL 8 and CXCL10), type 1 interferons (IFN-α, β)], pro-inflammatory cytokines [tumor necrosis factor alpha (TNF-α), IL-1β and IL-6], and even antimicrobial peptides (El-Zayat et al., 2019).

NLR classification and its activity is divided into four subfamilies based on their domains: an acidic domain—NLRA, a baculovirus inhibitor of apoptosis repeat (BIR) domain—NLRB, a caspase activation and recruitment domain (CARD)—NLRC, and a pyrin domain (PYD)—NLRP. With the exception of NLRX1 comprising a non-identified CARD-related X effector domain (Parlato and Yeretssian, 2014), NLRs are responsible for the formation of inflammasomes and lead to the activation of IRF, mitogen-activated protein kinase (MAPK), and NF-κB pathways initiating a robust immune response during pathogen attack (Zhong et al., 2013).

#### C-type lectin receptors and RLRs

C-type lectin-like domain proteins (CTLD) are CTLRs which function by binding glycans on the surface of microbial pathogens to regulate both innate and adaptive immune responses. Other CTLRs play a regulatory role in immune homeostasis by binding endogenous ligands (self-antigens; Mayer et al., 2017; Dierking and Pita, 2020). CTLRs contribute to the homing of immune cell-trafficking, leukocytes, pathogen recognition and subsequent T cell activation (Mayer et al., 2017). RLRs in turn are cytoplasmic sensors of viral RNA. RLR signaling leads to activation of players such as NF-κB, MAPK and IRFs which promote induction of type I IFN and some of the pro-inflammatory cytokines (Kawai and Akira, 2009; Lavelle et al., 2010).

#### G-protein coupled receptors and PGRPs

GPCRs and PGRPs are another group of human host receptors which recognize microbiota-derived signals. The GPCRs recognize the microbiome through a signal peptide or short chain fatty acids (Dierking and Pita, 2020). GPCRs such as FPR1 (formyl peptide receptor 1), FPR2, GPCR41 or FFAR3 (free fatty acid receptor 3) and GPCR43 or FFAR2 can detect bacterial MAMPs (Bloes et al., 2015). While FPRs carry important roles in inflammation and immune cell activation, FFARs are essential nutritional components which function as signaling molecules that regulate cellular and physiological processes (He and Ye, 2017; Kimura et al., 2020). On the other hand, the mammalian PGRPs have a regular antibacterial activity by contributing to the activation of macrophages during immune responses (Dierking and Pita, 2020).

### Polymorphisms of microbial sensors

Immuno-polymorphism is a significant aspect of infectious diseases biology that has been explored genetically in population-wide studies shedding light on the association of polymorphisms with epidemiology of several infectious diseases. Polymorphisms in the nucleotide or amino acid sequences of host receptors can influence downstream signaling pathways which in turn promote the resistance or susceptibility to various infections.

#### Polymorphism in TLRs and NLRs

A number of polymorphisms are directly associated with different phenotypes of infectious diseases of the gut (Table 1). TLR4 polymorphism (D299G and T399I) was reported as the first genetic variant in TLRs to reduce lipopolysaccharides-receptor interactions, which increased susceptibility to sepsis-inducing Gram-negative bacterial infections (Zhang et al., 2021). There are other common residues exhibiting single nucleotide polymorphisms (SNPs) in TLRs, namely, TLR1-I602S, TLR2-R677W, TLR3-P554S, TLR4-D299G, TLR5-L616F, TLR6-P249S, TLR7-Q11L, TLR8-M1V, TLR9-G1174A and TLR10-G1031T (Mukherjee et al., 2019). Polymorphisms in the TLR receptors are known to be associated with disease phenotypes such as ulcerative colitis, pancolitis, decreased or increased incidence and susceptibility to diseases such as inflammatory bowel disease and Crohn’s disease (CD; Mukherjee et al., 2019). CD is a chronic inflammatory bowel disease (IBD) which causes inflammation of the digestive tract mucosa with symptoms such as abdominal pain, fistulas, severe diarrhea, weight loss and malnutrition.

**Table 1:** Table showing important examples of human host receptors, their polymorphisms and their associated diseases.

Human host receptor	Category	Polymorphism	Disease condition	References
*Toll-like receptors (TLRs)*				
TLR1	Cell membrane TLR	R80T	Complicated skin and skin structure infection (cSSSIs), pancolitis, invasive aspergillosis	D’Onofrio et al., 2020, Stappers et al., 2014
TLR2	Cell membrane TLR	T597C	Tuberculosis meningitis	Thuong et al., 2007
TLR3	Intracellular TLR	L412F	Herpes labialis	Yang et al., 2012
TLR4	Cell membrane TLR	D299G	Inflammatory bowel disease (IBD)	Meena et al., 2013
TLR5	Cell membrane TLR	L616F	Crohn’s disease	Sheridan et al., 2013
TLR6	Cell membrane TLR	P249S	cSSSIs	Stappers et al., 2014
TLR7	Intracellular TLR	Q11L, Y264H	Hepatitis C virus (HCV), systemic lupus erythematosus	Schott et al., 2007, Brown et al., 2022
TLR8	Intracellular TLR	129C/G	Crimean-Congo hemorrhagic fever	Engin et al., 2010
TLR9	Intracellular TLR	1635A/G	HIV infection	Bochud et al., 2007
TLR10	Cell membrane TLR	N241H, I369L	Prostate cancer	Su et al., 2021
*NOD-like receptors (NLRs)*				
NOD2	Caspase activation and recruitment domain containing NLR	1,007 fs, R702W, G908R	Crohn’s disease	Sidiq et al., 2016
		L469F, R334Q, R334W, R702W, G908R	Blau syndrome, arthritis, gastrointestinal cancer	Caso et al., 2015 Liu et al., 2014
NLRP1	Pyrin domain containing NLR	M77T	Corneal intraepithelial dyskeratosis	Soler et al., 2013
		L155H-V1059M-M1184V	Autoimmune diseases	Levandowski et al., 2013
NLRP3	Pyrin domain containing NLR	See the source references	Inflammatory bowel diseases, alzheimer’s disease	Zhen and Zhang, 2019, Feng et al., 2020
NLRP7	Pyrin domain containing NLR	A719V, F671Glnfs*18	Abnormal human pregnancies and embryonic development	Soellner et al., 2017
NLRP12	Pyrin domain containing NLR	R284X, c.2072 + 3insT	Hereditary periodic fever syndromes, dermatitis	Wang, 2022
*G-protein coupled receptors (GPCRs)*				
CaSR	Calcium-sensing GPCR	L123S	Autosomal dominant hypocalcemia (ADH), Sporadic hypoparathyroidism, Familial hypoparathyroidism	Festas Silva et al., 2021
CXCR4	Chemokine GPCR	S339fs5	WHIM syndrome	Luo et al., 2022
EDNRB	Endothelin GPCR	W276C	Hirschsprung’s disease	Chatterjee and Chakravarti, 2019
FSHR	Glycoprotein hormone GPCR	A189V	Female infertility	Lundin et al., 2022
FPR1	Formyl peptide GPCR	F110S, C126W	Juvenile periodontitis	Jones et al., 2003
FZD4	Class frizzled GPCR	R417Q	Familial exudative vitreoretinopathy (FEVR)	Kondo et al., 2007
GNRHR	Goandotropin-releasing hormone GPCR	Q174R	Hypogonadotropic hypogonadism (HH)	Wang et al., 2021
GPR54	Kisspeptin GPCR	T305C	Hypogonadotropic hypogonadism (HH)	Alzahrani et al., 2019
GPR56	Adhesion class GPCR	R565W, L640R	Bilateral frontoparietal polymicrogyria (BFPP)	Chiang et al., 2011
LGR8	Relaxin family peptide GPCR	I604V	Cryptorchidism	Bogatcheva and Agoulnik, 2005
MASS1	Adhesion class GPCR	S2652X	Usher syndrome, Fibrile seizures (FS)	Nakayama et al., 2002
MC4R	Melanocortin GPCR	P78L	Dominant and recessive obesity	Wang et al., 2014
RHO	Opsin GPCR	P23H	Retinitis pigmentosa (RP)	Lee et al., 2021
AVPR2	Vasopressin and oxytocin GPCR	R113W	Nephrogenic diabetes insipidus (NDI)	Cheong et al., 2007
ß1 Adrenergic receptor	Andrenoceptors	R389G	Heart failure	Zhang and Steinberg, 2013
ß3 Adrenergic receptor	Andrenoceptors	W64R	Obesity	Haji et al., 2021

NLR mutations on the other hand, are quite often associated with syndromes of autoinflammatory mechanisms, hinting at a convoluted role of cytosolic surveillance in systemic innate immunity (Delbridge and O’Riordan, 2007; Table 1). NOD1 and NOD2 are PG sensors which specifically respond to the PG components found in the rigid cell wall of the bacteria. A genetic association between NOD1 and NOD2 mutations and autoinflammatory diseases shows the importance of NOD proteins in inflammation regulation (Hugot et al., 2001; Girardin et al., 2005; McGovern et al., 2005). However, a direct contribution of NOD proteins to patients with immunodeficiency is not yet established (Delbridge and O’Riordan, 2007). NOD2 with its three coding variants (R702W, G908R, and L1007fs) resulting in decreased muramyl-dipeptide (MDP) responsiveness has been associated with a strong genetic risk factor for development of CD (Parlato and Yeretssian, 2014). On the other hand, genetic studies on NOD1 variants with susceptibility to IBD need further investigation due to conflicting information in literature (McGovern et al., 2005; Tremelling et al., 2006). The implications of NOD1 and NOD2 in bacterial sensing, primarily through the strong association of NOD2 with CD, has been under speculation that an altered detection of commensal microbes might lead to the impairment of homeostasis in the intestines thereby leading to intestinal inflammation (Parlato and Yeretssian, 2014).

#### Polymorphism in CTLRs and RLRs

Multiple CTLRs and RLRs variants have been reported in literature against non-enteric infectious microbes. In CTLRs, two variants, G54D (rs1800450) and N104S (rs2617170) in MBL2 and KLRC4, respectively, contribute to the susceptibility of Behcet’s disease (Yang et al., 2017). Behcet’s (beh-CHETS) disease is an autoimmune disease that causes systemic blood vessel inflammation. In particular, G1186A mutation in a CTLR known as MRC1 (mannose receptor 1) was linked to an increased risk of pulmonary tuberculosis among the Chinese population (Zhang et al., 2012). Recently, four CLEC4E SNPs (rs10841845, rs10841847, rs10841856, and rs4620776) were identified to be a cause of pulmonary tuberculosis (Bowker et al., 2016). One such finding reports broad spectrum MBL2 polymorphisms associated with human immunodeficiency virus (HIV) and tuberculosis co-infection across diverse populations (Garcia-Laorden et al., 2006). In RLRs, mutations at residues threonine 770, serine 854 and serine 85, were shown to constitutively activate RIG-I resulting in IFN induction (Sun et al., 2011). A non-synonymous polymorphism due to mutation of arginine to cysteine at amino acid position 7 leads to the expression of a functional RIG-I associated with an increased antiviral signaling (Shigemoto et al., 2009; Hu et al., 2010; Loo and Gale Jr, 2011).

#### Polymorphism in GPCRs and PGRPs

GPCRs, like TLRs and NLRs play an important role in the recognition of MAMPs. The transcription in cells may be affected in a tissue-selective manner which in turn may be affected by the polymorphisms of the GPCR promoter regions genes (Insel et al., 2007; Table 1) while genetic variants in PGLYRP1, PGLYRP2, PGLYRP3 and PGLYRP4 genes were shown to be associated with CD and ulcerative colitis (Zulfiqar et al., 2013; Dierking and Pita, 2020).

### Microbial ligands

Microbial metabolites are chemical compounds which have the ability to regulate host immune responses by activating human host receptors (Rooks and Garrett, 2016). On the other hand, microbial ligands which bind to the receptors as mentioned above, also referred to as the PAMPs recognized by PRRs, can be grouped into three main classes, i.e., glycans, nucleic acids, and the proteins (Kawai and Akira, 2009). These microbial ligands do not share structural similarity but they share features which reflect the evolutionary progression of response to innate immunity. First, these are produced by the microbe (commensal/ pathogenic) and not by the host. This is the basis of discrimination of the self and non-self-antigens which in turn enables the host to mount an innate or adaptive immune response against microbial agents. Second, these ligands are conserved among a certain class of pathogens (Rana et al., 2015). Hence, recognition of a conserved portion of the lipopolysaccharide (LPS) lets PRR to identify the presence of any Gram-negative bacteria. Third, as these PAMPs carry out functions which are physiologically essential for microbial survival, any mutation in PRRs would be toxic or detrimental to the host itself (Medzhitov and Janeway Jr, 2000; Mogensen, 2009).

#### Microbial effector proteins

Microbial effector proteins are known to encompass catalytic domains within their primary sequences and exhibit enzymatic activities (Popa et al., 2016). Effector proteins are classified into four main categories based on the mode of action on the host (Scott and Hartland, 2017): 1. As competitive inhibitors which directly bind with host receptors (NleF—*Escherichia*, VipD—*Legionella*), 2. As proteins which functionally mimic the host proteins by post translational modifications (PTMs) such as lipidation (SopE—*Shigella*, SifA—*Salmonella*), 3. As mediators of PTM to block host protein functions (AnkX—*Legionella*, OspF—*Shigella*), and 4. As proteolytic enzymes which alter the host proteome composition and its spatial organization (NleC—*Escherichia*, RavZ—*Legionella*; Paul, 2012). Effector proteins expressed by different bacteria modify host cell proteins aiming to suppress the host defense mechanisms. This in turn allows the pathogens to source essential nutrients from the host and cause infection. Bacterial effector proteins are reported to be distinctively associated with different receptors and activate different downstream signaling pathways—thus establishing the differences between commensals and pathogens (Weigele et al., 2017; Mak and Thurston, 2021).

Among the various functions of the effector proteins, the ability to alter the host protein signaling pathways using PTMs is the most interesting one from a therapeutic perspective (Forrest and Welch, 2020). Pathogenic bacteria, both intracellular and extracellular, secrete effector proteins into the host cell which intervenes in the immuno-modulatory response by mimicking the natural process in the host cell. Among the modifications are phosphorylation, acetylation, methylation, ribosylation, adenylation, lipidation, glycosylation, ubiquitination and the reversal of these modifications (Forrest and Welch, 2020). Irreversible changes like proteolysis, eliminylation and deamidation also perturb the host response pathway. Given the fact that the actual modification occurs due to enzymatic reaction by the effector proteins, the therapeutic interventions in these pathways find a spotlight as potential alternative treatment(s), especially against drug-resistant infections (Stévenin and Neefjes, 2022). Along with the host protein signaling pathways, the host receptors themselves go through a series of PTMs whose role in pathogenesis, if further deciphered would expand our understanding of the molecular and cellular basis of innate immunity (Liu et al., 2016; Patwardhan et al., 2021).

Phosphorylation is a well-known PTM in which a phosphoryl group is transferred to a hydroxyl group of serine, threonine or tyrosine residues and is critical to host cell functions. Infectious bacteria exploit this pathway by secreting their own (de)phosphorylating enzymes to alter the host cell response. For example, in entero-haemorrhagic *E. coli* (EHEC) O157:H7 (Hua et al., 2020), the effector protein EspF interacts with annexin A6 (ANXA6) of the host protein resulting in phosphorylation of myosin light chain (MLC) and activation of protein kinase C (PKC) leading to the disruption of tight junctions between host cells, thus weakening the intercellular integrity. Lysine acetylation and methylation are other well-known PTMs where an acetyl group or a methyl group is transferred, respectively, to the epsilon-amino group in the side chain of a lysine residue in a reversible manner. In contrast, invasive pathogens such as *Salmonella* hijack their own process *via* acetylation to mediate and downregulate the effector proteins (Sang et al., 2016). In the case of *Shigella*, the effector protein OspZ methylates cysteines in NF-kB activators TAB2 and TAB3 preventing the release of pro-inflammatory cytokine IL-8 (Zheng et al., 2016). In contrast to addition of chemical groups on to the host proteins, ubiquitination (and ubiquitin-like protein conjugation) involves the addition of a small ubiquitin (or ubiquitin-like) protein on to the amino-group of a lysine residue or the N-terminal methionine residue in the host protein. A concerted action of ubiquitinating enzymes facilitate this process while proteases called deubiquitinases (DUBs) reverse the ubiquitination. Several enteric pathogens are known to hijack this process to modulate the immune response. For example, *Salmonella* uses SseL for deubiquitinating polyubiquitin chains (Rytkönen et al., 2007) while AvrA removes ubiquitin from nuclear factor of kappa light polypeptide gene enhancer in B-cells inhibitor, alpha (IκBα) and β-catenin resulting in the regulation of the NF-κB and β-catenin signaling pathways (Ye et al., 2007). In some cases, the bacterial effector proteins also mimic the ubiquitinating enzymes of the host protein. Such proteins are called Novel E3 ligases (NELs). For example, *Shigella* secretes NEL called IpaH9.8 which contributes to the splicing of U2AF35 mRNA resulting in the suppression of host immune response (Rytkönen et al., 2007).

### Possible targets for discovery

**Figure 1:**
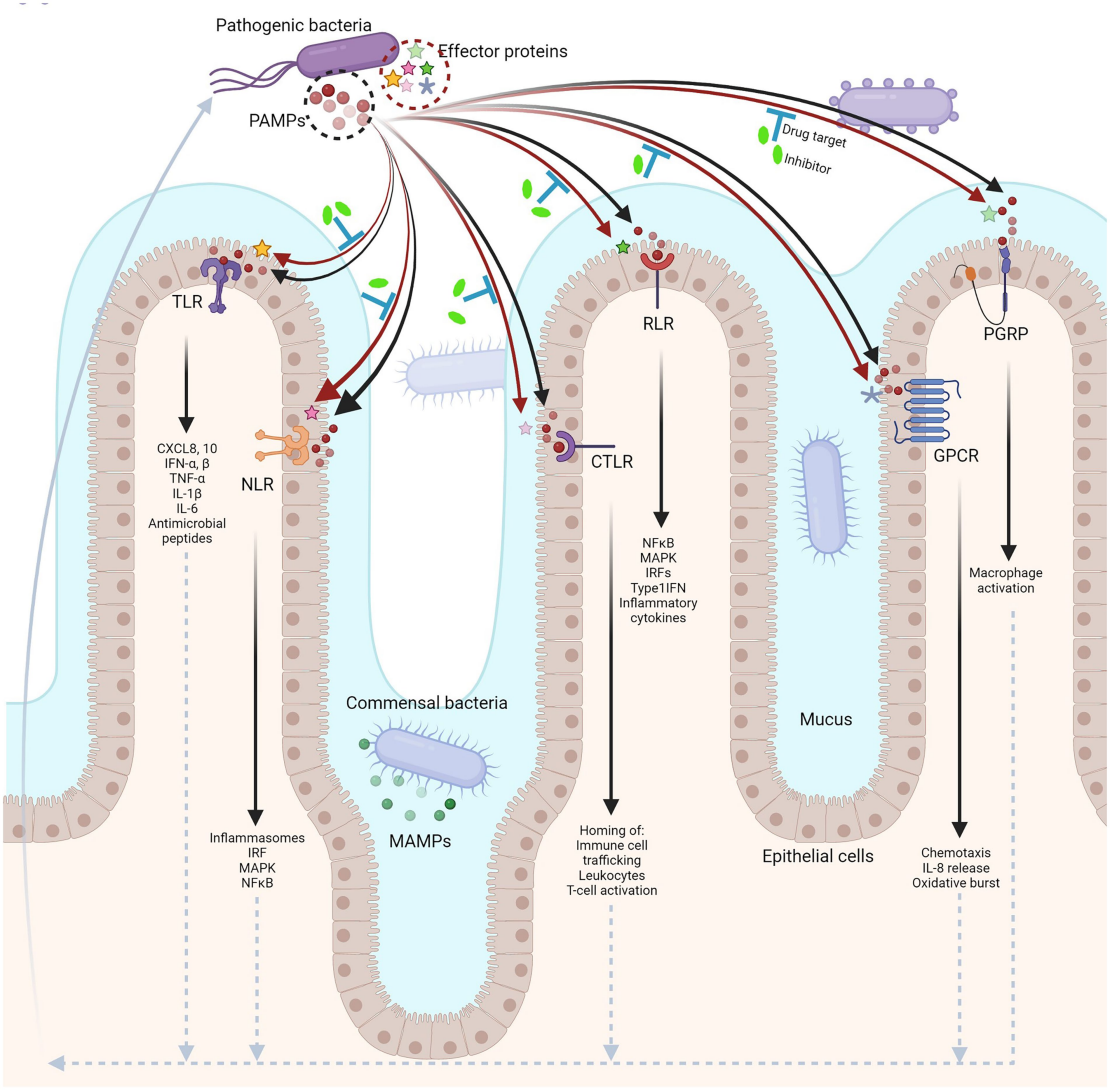
Host–microbe interface in the human gut. Different pattern recognition receptors (PRRs) in the host distinctly recognize PAMPs from pathogenic bacteria (black arrows), eliciting an immune response (upward arrow at far left, dotted arrows). In turn, pathogens secrete effector proteins that can modulate the pathways involved in host-immunity (red arrows). The enzymatic functions of effector proteins can be targeted for drug interventions contributing to alternative therapies against infections (blue ‘T’ bars; created with BioRender.com).

Given the myriad of host receptor proteins modulated by pathogenic effector proteins in an enzymatic fashion, such processes are considered high profile therapeutic targets for drug interventions (Figure 1). The emergence of antibiotic resistance, compounded by the growth-lag in developing new antibiotics also necessitates the identification of alternative targets for antimicrobial therapy. In this aspect, new avenues are constantly being explored. We have discussed the important interactions in the previous section. The underlying biochemical mechanisms of such interactions and the PTMs associated with pathogenesis could potentially unlock the therapeutic potential of targeting these enzymes. Though in its infancy, the existing state-of-the-art high throughput screening (HTS) facilities and the ever-expanding repertoire of chemical inhibitors and small molecule libraries has pushed the frontiers in the struggle to control enteropathogenic infections with the help of new therapeutic molecules.

Some of the biochemical pathways involved in infection have been extensively studied to identify small molecule inhibitors that can influence the activity of PTM-promoting effector proteins. For example, *Mycobacterium* tyrosine phosphatases namely mPTPA and mPTPB were targeted for identifying inhibitors that can potentially lead to therapeutic application (Ruddraraju et al., 2020). Such techniques can be applied to the effector proteins identified in several species of enteric bacteria using similar screening techniques.

There are several mechanisms through which pathogenic bacteria develop drug resistance, including drug inactivation, modification of bacterial target sites, reduced antibiotic uptake and the formation of biofilms. These mechanisms explain how WHO “priority status” listed ESKAPE pathogens (*Enterococcus faecium*, *Staphylococcus aureus*, *Klebsiella pneumoniae*, *Acinetobacter baumannii*, *Pseudomonas aeruginosa*, and *Enterobacter* species) have developed fitness against a wide-range of compounds such as lipopeptides, fluoroquinolones, tetracyclines, β-lactams and antibiotics that are the last line of defense (De Oliveira et al., 2020). One way to circumvent this problem is to use a multi-drug target instead of a single target thereby overcoming the susceptibility to resistance due to mutations. Single drug-target systems could be prone to drug resistance due to mutations (evolutionarily unavoidable). Therefore, multiple drug-target systems linked by a chemical linker are more effective. For example, in *Helicobacter*, using a multi-drug system such as the quadruple therapy is effective given that development of multiple phenotypic resistance is often difficult and slow. Similarly, targeting homomers with multiple binding sites (MBS) is better than targeting a single binding site (SBS; Abrusán and Marsh, 2019). Another avenue that is recently being explored is the host-directed therapy (HDT). HDT involves interfering with host factors important for pathogens to invade and cause disease (Ruddraraju et al., 2020). This can be carried out in two steps: directly interfering with host response pathways and enhancing immune response to infection (Kaufmann et al., 2018; Abrusán and Marsh, 2019). Furthermore, advances in the ‘omics’ research of the gut-microbe interactions have opened up possibilities of finding additional targets for drug discovery. Most of these interactions are followed up using mass spectrometric techniques using samples from diseased patients. Such data are consolidated into an interaction map providing a holistic approach for both diagnosis and treatment for various gut-related diseases (Lloyd-Price et al., 2019; Whon et al., 2021).

In addition to this, SNPs are known to be one of the major elements associated with the fate of a microbial infection (Mukherjee et al., 2019). Studies conducted in a German cohort showed correlation of SNPs, G743A and T1805G with cell surface TLR1 expression deficiency resulting in susceptibility to *M. tuberculosis* infection. Two additional SNPs, S150G and V220M which are associated with the structural organization of the LRR motif of TLR1 further refined the understanding of susceptibility to *M. tuberculosis* in the context of receptor recognition ability of mycobacteria (Bustamante et al., 2011; Uciechowski et al., 2011). Another interesting observation reported in Asian patients having acute febrile malaria demonstrates the presence of polymorphisms (rs5743551) on G allele of TLR1 leading to reduced parasitemia and thereby a host directed control on severity of the disease (Hahn et al., 2016). From an evolutionary thinking, this points to the possibility that G allele of *TLR1* rs5743551 occurs in higher frequencies in non-Caucasian hosts and imparts a fitness advantage in malaria endemic zones. The state-of-the-art CRISPR/Cas9-based genome editing could be employed to target the microbes which cause pathogenesis in the host (Zheng et al., 2022). Polymorphisms in host receptors can further be exploited to understand the virulence potential of pathogenic bacteria that can be targeted for suppression by CRISPR-Cas9 based therapies (Ramachandran and Bikard, 2019; Nath et al., 2022).

## Conclusion

Sustained efforts directed at understanding the gut microbiome and the immune interactions have contributed to our understanding of the role of dysbiosis in the immune system concerning a range of infections and disease phenotypes. However, due to a relentless rise in antibiotic resistance, the human gut, which is home to the largest ecosystem of microbes, is at risk of becoming a hot-zone of a wide range of potential infections and pathologies. In future, alternative therapies are critical to replace current infection control regimens by targeting the receptor-ligand interactions and their downstream processes in the gut. In this regard, it is important to understand how commensal and pathogenic bacteria interact with the host in order to maintain symbiosis or trigger an infection. A few interesting aspects need to be addressed in relation to the axis of host-pathogen-environment: colonization of the mucus layer by commensal bacteria while in symbiosis with the host in the presence of specific MAMPs; existence of commensal MAMPs alongside pathogenic variants which can be distinguished from each other based on their environment; and restoring commensality to the pathogenic PAMPs by altering the host environment. Considering the fact that even a single polymorphism might facilitate a commensal-to-pathogen transition, it is important to develop a holistic approach towards an assay platform that profiles pathogenic PAMPs and the commensal MAMPs based on molecular, physiological and host genetic factors. This can be exploited in developing molecular screens targeting the host-pathogen interface in addition to better understanding the relationship between microbial sensors and enteric bacteria and how they affect susceptibility or resistance to an infection.

## Author contributions

ST and DSH contributed to writing of the review and editing. NA contributed to narrative building, discussion and editing. All authors contributed to the article and approved the submitted version.

## Conflict of interest

The authors declare that the article was compiled in the absence of any commercial or financial relationships that could be construed as a potential conflict of interest.

## Publisher’s note

All claims expressed in this article are solely those of the authors and do not necessarily represent those of their affiliated organizations, or those of the publisher, the editors and the reviewers. Any product that may be evaluated in this article, or claim that may be made by its manufacturer, is not guaranteed or endorsed by the publisher.
